# Uric acid, high density lipoprotein cholesterol levels and their ratio are related to microbial enterotypes and serum metabolites in females with a blood stasis constitution

**DOI:** 10.1186/s12944-024-02066-4

**Published:** 2024-03-27

**Authors:** Chen Sun, Yanmin Liu, Wei Huang, Yang Chen, Yusheng Deng, Jiamin Yuan, Lili Deng, Ning Xu, Xiaoxiao Shang, Chuyang Wang, Zhimin Yang, Li Huang, Qinwei Qiu

**Affiliations:** 1 State Key Laboratory of Dampness Syndrome of Chinese Medicine, The Second Affiliated Hospital of Guangzhou University of Chinese Medicine (Guangdong Provincial Hospital of Chinese Medicine), Guangzhou, China; 2 The Second Affiliated Hospital , Guangzhou University of Chinese Medicine (Guangdong Provincial Hospital of Chinese Medicine), Guangzhou, China; 3 Guangdong Provincial Hospital of Chinese Medicine, Fangcun Hospital, Guangzhou, China

**Keywords:** Traditional Chinese medicine, Blood stasis, Gut microbiome, Serum metabolome, High-density lipoprotein cholesterol, Uric acid

## Abstract

**Background:**

Blood stasis constitution in traditional Chinese medicine (TCM) is believed to render individuals more susceptible to metabolic diseases. However, the biological underpinnings of this constitutional imbalance remain unclear.

**Methods:**

This study explored the association between blood stasis constitution, serum metabolic markers including uric acid (UA), high-density lipoprotein cholesterol (HDLC), their ratio (UHR), serum metabolites, and gut microbiota. Clinical data, fecal and serum samples were acquired from 24 individuals with a blood stasis constitution and 80 individuals with a balanced constitution among healthy individuals from Guangdong. Gut microbiota composition analysis and serum metabolomics analysis were performed.

**Results:**

Females with a blood stasis constitution had higher UA levels, lower HDLC levels, and higher UHR in serum, suggesting a higher risk of metabolic abnormalities. Analysis of the gut microbiome revealed two distinct enterotypes dominated by *Bacteroides* or *Prevotella*. Intriguingly, blood stasis subjects were disproportionately clustered within the *Bacteroides*-rich enterotype. Metabolomic analysis identified subtle differences between the groups, including lower phenylalanine and higher trimethylaminoacetone levels in the blood stasis. Several differential metabolites displayed correlations with HDLC, UA, or UHR, unveiling potential new markers of metabolic dysregulation.

**Conclusions:**

These findings elucidate the intricate interplay between host constitution, gut microbiota, and serum metabolites. The concept of blood stasis offers a unique perspective to identify subtle alterations in microbiome composition and metabolic pathways, potentially signaling underlying metabolic vulnerability, even in the presence of ostensibly healthy profiles. Continued investigation of this TCM principle may reveal critical insights into the early biological processes that foreshadow metabolic deterioration.

**Supplementary Information:**

The online version contains supplementary material available at 10.1186/s12944-024-02066-4.

## Background

Uric acid (UA) and high-density lipoprotein cholesterol (HDLC) are two indispensable biomarkers that play pivotal roles in the metabolic processes of the human body. UA, the ultimate product of purine metabolism, protects against oxidative stress-related conditions. Nevertheless, elevated levels of serum UA, known as hyperuricemia, contribute to the development of gout [1], hypertension [2], renal disease, and metabolic syndrome [3]. HDLC is a crucial constituent of the lipid profile and contributes to reverse cholesterol transport, which collects cholesterol from peripheral tissues and restores it back to the liver for excretion. Elevated serum HDLC levels correlate with reduced risk of atherosclerosis and cardiovascular disease, as it is believed to exhibit anti-inflammatory, antioxidative, and antithrombotic properties [4, 5]. Conversely, low HDLC levels independently increase the risk of metabolic syndrome and related disorders [6]. The UA to HDLC ratio (UHR) has recently emerged as a potentially valuable clinical indicator of metabolic abnormalities. Increasing evidence suggests that an increased UHR is associated with heightened risks of chronic kidney disease [7], insulin resistance [8, 9], metabolic syndrome [10, 11], and cardiovascular disease [12, 13], underscoring its potential as a valuable adjunct to conventional risk factors in identifying individuals at higher risk of developing metabolic disorders.

Human metabolism is significantly impacted by the gut microbiota. Many metabolic diseases, such as obesity, diabetes, and cardiovascular disorders, are linked to gut microbiome dysbiosis [14]. Certain gut microbiota deviations, such as short-chain fatty acids, bile acids and trimethylamine N-oxide, are thought to be key elements that help regulate the host’s physiological processes [15]. The linkages between the gut microbiota and serum UA and HDLC levels are being established. Compared to individuals with normouricemia, those with hyperuricemia exhibit decreased richness and diversity of their microbiota, along with changes in the composition of the *Coprococcus* genus, which is less prevalent in individuals with hyperuricemia [1]. Manipulation of the gut microbiota through prebiotics or probiotics may help reduce serum UA levels by promoting purine and UA catabolism, increasing UA excretion, regulating UA absorption or secretion in the intestinal tract, and mitigating the intestinal inflammatory response to the gut microbiota [16, 17]. Similarly, the gut microbiome influences lipoprotein cholesterol metabolism through multiple pathways, including those involving the aforementioned compounds [18]. Interestingly, recent studies have shown that HDLC is synthesized by the intestine rather than just the liver [19], and cholesterol dehydrogenases have been identified from gut microbiomes to reduce the host intestine and serum cholesterol levels [20].

Constitution of traditional Chinese medicine (TCM) is a classification system based on individual health, which is determined by the environment, lifestyle, genetic makeup and other factors [21]. According to TCM philosophy, a balanced constitution reflects a state of complete well-being, whereas distinct imbalanced constitutions exhibit differential vulnerability to illnesses and impact the progression and prognosis of diseases. Recent advances in omics have supported the biological basis of constitution categorization. Researchers have found that certain genetic variations and protein expression levels are associated with specific constitutions [22]. Among the biased constitutions, the blood stasis is believed to be correlated with poor blood circulation and to render individuals more susceptible to metabolic diseases such as diabetes mellitus, hypertension, heart disease, cholelithiasis, and hyperlipidemia [22]. However, the biological characteristics of the blood stasis, including clinical observational indexes and omics features, remain unclear.

We hypothesized that the constitution classification in TCM, specifically the presence of blood stasis, may be related to distinct characteristics in the gut microbiome and serum metabolome. Exploration of the linkages between TCM constitutions, gut microbiota, and serum metabolites, could help explain the potential biological underpinnings of a blood stasis and the intricate interactions among these factors in the context of metabolic health. Therefore, this study compared and analyzed the microbiome and serum metabolome in individuals with a balanced constitution to those with a blood stasis constitution. The results revealed several differential features associated with serum UA, HDLC, and/or UHR levels. These findings have significant implications for understanding the biological mechanisms underlying blood stasis and the complex interactions among the gut microbiota, serum metabolites, and constitutional imbalances.

## Method

### Study design and ethical approval

Following the National Standards of Classification Balance and Determination of Constitution of Chinese Medicine issued by the China Association of Chinese Medicine [23], 24 blood stasis subjects and 80 balanced subjects were collected from the cohort of Guangdong Provincial Hospital of Chinese Medicine, Fangcun Hospital. All participants were aged 18 to 60 years and were in good health. In addition to the clinical information obtained from physical examination and fasting blood tests, fecal samples were acquired for gut microbiome analysis, and serum samples were obtained for metabolome analysis. This study was approved by the Human Research Ethics Committee of Guangdong Provincial Hospital of Chinese Medicine (ethical review number: B2017-199-01), and the approved guidelines were followed for all methods. Informed written consent was acquired from all participants.

### Fecal DNA extraction and metagenomics sequencing

Using a Magnetic Soil and Stool DNA Kit (TIANGen, DP812), DNA was extracted from feces. The degree of contamination and degradation of the DNA samples was observed via 1% agarose gel electrophoresis. A Qubit dsDNA Assay Kit and Qubit 2.0 Fluorometer (Life Technologies, CA, USA) were employed to measure the DNA concentration. Only samples with an optical density value between 1.8 and 2.0 and a DNA content exceeding 1 µg were utilized for library construction. The NEBNext Ultra II TM DNA Library Prep Kit for Illumina (NEB, USA) was used to prepare sequencing libraries, and index codes were used to assign sequences to the samples. In brief, the DNA was sonicated for fragmentation, resulting a size of 350 bp. Subsequent steps included end-polishing, A-tailing, ligation with the full-length adaptor, and PCR amplification. The products were ultimately purified using the AMPure XP system, and the distribution of library size was assessed by Agilent 2100 Bioanalyzer. Real-time PCR was used for quantification. As directed by the manufacturer, index-coded samples were clustered using a cBot Cluster Generation System. Following the generation of clusters, the prepared libraries were sequenced on the Illumina NovaSeq 6000 platform to produce paired-end reads.

### Bioinformatics and statistical analysis of microbiota data

The biobakery3 workflows [24] were utilized to process the raw sequencing files. Human DNA sequences were initially identified and eliminated with Kneaddata v0.10.0 (https://huttenhower.sph.harvard.edu/kneaddata). Subsequently, microbial taxonomic profiles were generated using MetaPhlAn3, while functional profiling of microbial pathways was characterized with HUMAnN3. The species richness, Shannon index and Simpson index were calculated for alpha diversity assessment. Enterotype analysis was performed employing the Jensen‒Shannon divergence distance and clustering algorithm based on partitioning around medoids. The association between metadata and relative abundance was investigated by using MaAsLin2 [25]. Before MaAsLin2 analysis, metadata collinearity was checked using the Pearson correlation coefficient. If a Pearson’s |ρ| > 0.8 was obtained, collinearity was assumed, and the collinear variables were deemed redundant. One variable from each pair was then removed from further analysis. Differences in microbial characteristics were examined using linear discriminant analysis effect size (LEfSe) from R package microbiomeMarker [26], with a discriminant score LDA = 2 as a threshold.

### Serum metabolite extraction and untargeted metabolomic measurements

Metabolomics tests were conducted at Shanghai Biotree Biotech Co. Ltd. Briefly, after transferring 100 µL of each serum sample to an EP tube, and an additional 400 µL of extraction solution (consisting of acetonitrile methanol at a 1:1 ratio with an isotopically labeled internal standard mixture) was added. The specimens were agitated using a vortex for a duration of 30 s, followed by sonication in an ice-water bath for 10 min and incubated at -40℃ for 1 h for protein precipitation. Subsequently, the samples underwent centrifugation at a velocity of 12,000 revolutions per minute (RCF = 13,800 (×g) and *R* = 8.6 cm) for a duration of 15 min at a temperature of 4 degrees Celsius. The resulting liquid above the sediment was moved to a new glass container for examination, while an equal portion of the liquids from each of the samples was combined to create the quality control sample.

The Vanquish UHPLC (Thermo Fisher Scientific) was utilized for LC‒MS/MS analyses, in conjunction with an Orbitrap Exploris 120 Mass Spectrometer (Orbitrap MS, Thermo) and a UPLC BEH Amide column (2.1 mm × 100 mm, 1.7 μm). Solution A (25 mM ammonium acetate and 25 mM ammonia hydroxide dissolved in water at a pH of 9.75) and solution B acetonitrile constituted the mobile phase. The samples were kept in the autosampler at 4 °C, with 2 µL injected for each run. The data were acquired in IDA mode with Xcalibur software (version 4.4). The ESI source parameters were 50 Arb for the sheath gas flow rate, 15 Arb for the auxiliary gas flow rate, 320℃ for the capillary temperature, 60,000 for the full MS resolution, 15,000 for the MS/MS resolution, and 10/30/60 for the collision energies in NCE mode. For positive mode, the spray voltages were set at 3.8 kV and were adjusted to -3.4 kV for negative mode.

### Bioinformatics and statistical analysis of metabolomic data

Using the ProteoWizard toolkit, the raw data were subsequently transformed into the mzXML format. The data preprocessing included peak detection, extraction, alignment, and integration, which were implemented using an in-house R program based on XCMS. The annotation of the metabolites detected was carried out by an in-house MS2 database called Biotree DB, with unassigned metabolites filtered out before downstream analysis. Ultimately, 586 out of 10,421 metabolites in positive mode and 240 out of 8937 in negative mode were subjected to subsequent analysis, and their levels were corrected against internal standards. Principal component analysis (PCA) was employed to dimensionally reduce the metabolome profile, ensure analytical system stability, and identify the dataset’s groupings, trends, and outliers. Differential metabolites were selected via the partial least squares method-discriminant analysis (PLS-DA) considering a combination of variable importance in projection (VIP) values higher than 1 and *P* value < 0.05, by two-tailed Welch test.

### Bioclinical variable assays and statistical analysis

For the anthropometrics and biochemical indexes, Student’s t-test or ANOVA was employed for comparing normally distributed continuous variables, while the Mann‒Whitney U test was employed to analyze nonnormally distributed continuous variables. The chi-squared test or Fisher’s exact test was employed to analyze categorized variables. UHR was calculated by converting the UA value from µmol/L to mg/dL. All statistical calculations were conducted using R v4.22. Data visualization was conducted using the R packages ggplot2 and ggstatsplot [27]. *P* value < 0.05 was considered significantly significant.

## Results

### Blood stasis was associated with higher UA and lower HDLC levels in females

**Fig. 1:**
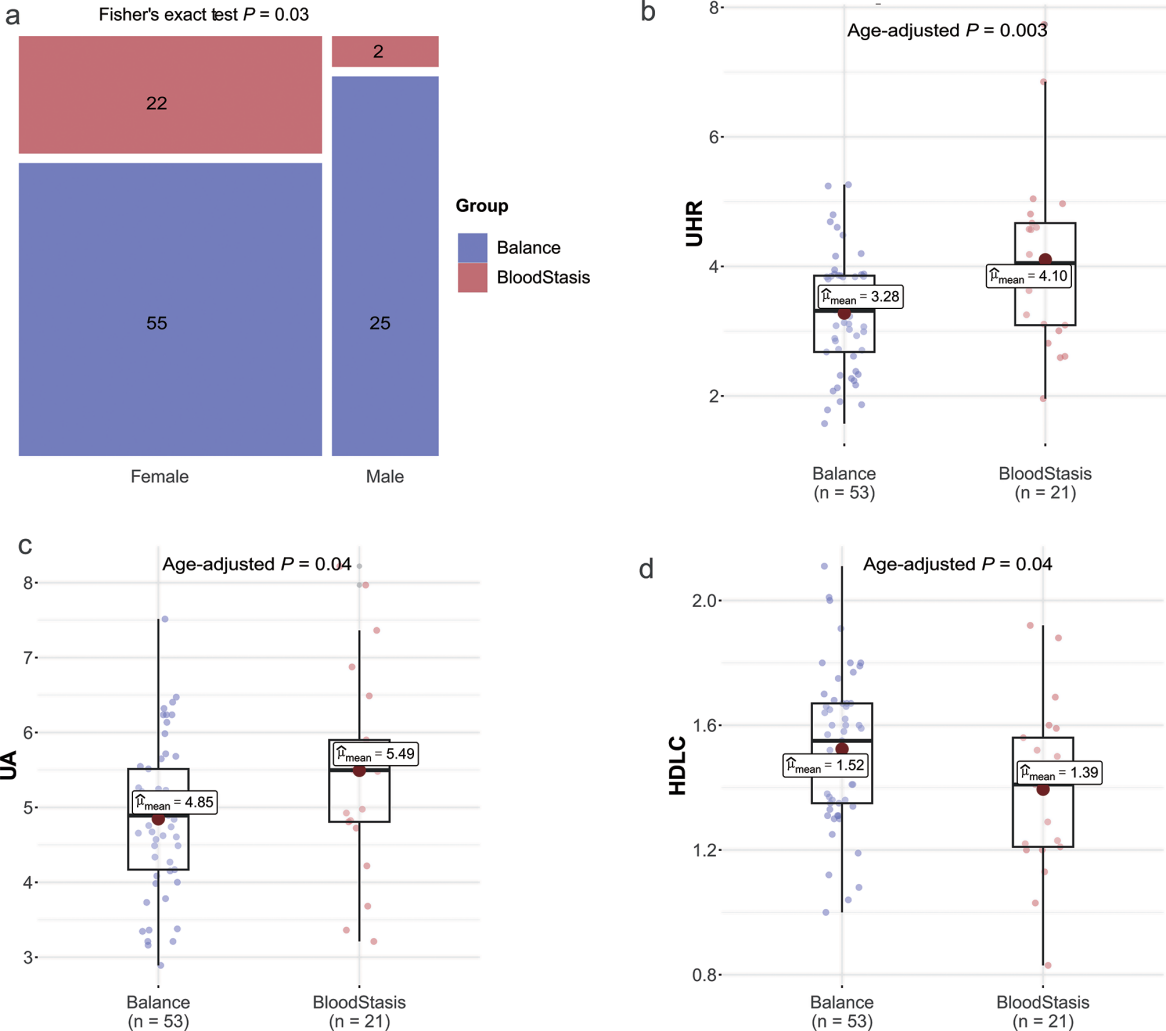
Differences in phenotypes between the blood stasis and balanced constitution groups. (**a**) Distribution of sexes in the constitution groups. Statistical significance was calculated using Fisher’s exact test. (**b–d**) The boxplots of (**b**) UHR, (**c**) UA, and (**d**) HDLC between the blood stasis and balanced group in females. Two balanced individuals and one blood stasis individuals with missing information on these indexes were not included in the analysis. Statistical significance was calculated using two-way ANOVA corrected for age

Under the National Standards of Classification Balance and Determination of Constitution of Chinese Medicine [23], 24 individuals with blood stasis and 80 with a balanced constitutions were identified. Population’s clinical parameters were detailed in Additional File 1, Table S1. The age range of these participants was 26–51 years, and all were deemed healthy based on their physical examination indicators and serum biochemical profile. Except for sex, no statistically significant differences existed in other indicators between the blood stasis and balanced constitution groups (see Additional File 1, Table S1, Fig. 1a). Notably, only two males were found to have blood stasis constitution. Considering the variations in reference intervals for indicators such as UA between sexes, and the observed association between age and various metabolic markers, a comparative analysis was conducted between blood stasis and balanced constitution groups in females, employing both age-adjusted and non-age-adjusted methods. These findings showed that females with a blood stasis constitution had significantly higher mean UA levels (5.49 µmol/L vs. 4.85 µmol/L, age-adjusted *P* value = 0.04) and significantly lower mean HDLC levels (1.39 µmol/L vs. 1.52 µmol/L, age-adjusted *P* value = 0.04) than females with a balanced constitution (Fig. 1b–c). The UHR was significantly higher in females with a blood stasis constitution than in those with a balanced constitution (t-test *P* value = 0.02; age-adjusted *P* value = 0.003) (Fig. 1d). These results suggested that females with blood stasis constitution may exhibit a higher risk of developing metabolic abnormalities, characterized by higher UA levels, lower HDLC levels, and elevated UHR in serum.

### Two enterotypes in females reflected differences in UA, HDLC, UHR, and blood stasis constitution

**Fig. 2:**
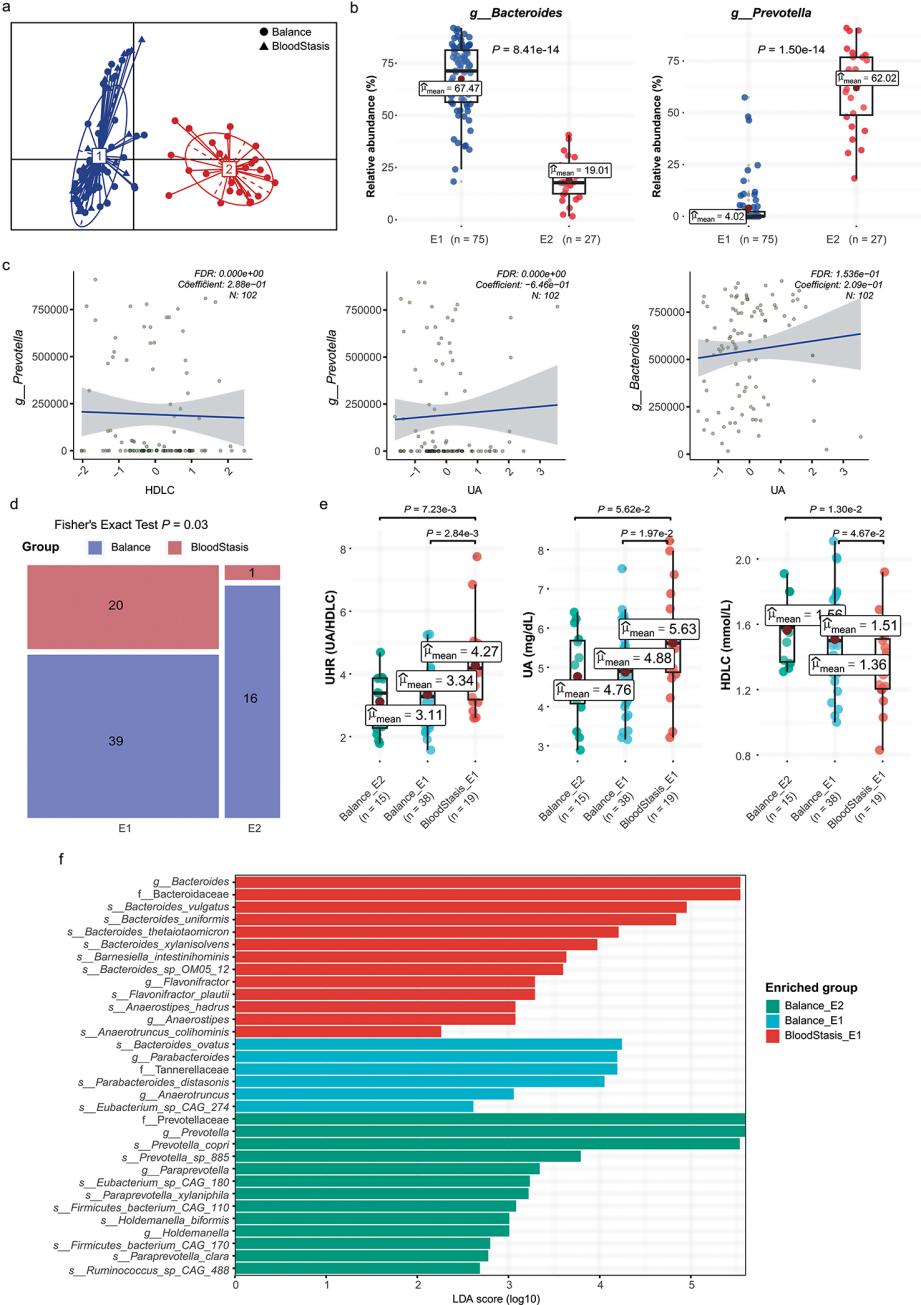
Results of gut microbiome analysis. (**a**) Samples colored by enterotype. Dark blue indicates enterotype 1 and crimson red indicates enterotype 2. (**b**) Relative abundance of *Bacteroides* and *Prevotella* in two enterotypes. The mean relative abundances were indicated as dark red points with labels. *P* values were determined by t-test. (**c**) MaAslin2 analysis confirmed the associations of *Bacteroides* abundance and *Prevotella* abundance with UA and HDLC. (**d**) Distribution of enterotypes in the female constitution groups. (**e**) The boxplots of UHR, UA, and HDLC across Balance_E2 (caribbean green), Balance_E1 (sky blue), and BloodStasis_E1 (vermilion) in females. *P* values were determined by t-test. There was only one sample in BloodStasis_E2 group, so it was not included in the analysis here. (**f**) LEfSe identified taxons for Balance_E2, Balance_E1, and BloodStasis_E1 in females. There was only one sample in the BloodStasis_E2 group, so it was not included in the analysis here

The gut microbiota characteristics of all samples were investigated except for two individuals (one from the balanced group and one from the blood stasis group) for whom fecal samples were unavailable. The gut microbiome compositions of all samples were similar at the phylum and class levels, with Bacteroidetes and Firmicutes being the most abundant phyla and Bacteroidia and Clostridia being the dominant classes (see Additional File 2, Fig. Sa-b). At the genus and species levels, however, the microbiome composition varied substantially between individuals. Most individuals were dominated by *Bacteroides* or *Prevotella*, and the most frequent species included *Bacteroides vulgatus, Prevotella copri*, *Bacteroides plebeius*, *Bacteroides uniformis*, *Bacteroides stercoris*, *Bacteroides dorei*, *Alistipes putredinis* and *Faecalibacterium parusnitzii*(see Additional File 2, Fig. Sc-d). A comparison of the gut microbiota characteristics between the blood stasis and balanced constitution groups revealed no significant differences in alpha-diversity indexes (richness, Shannon, and Simpson) in either the whole cohort or only female participants only (Additional File 3, Fig. Sa-b,). Next, the samples were clustered based on species-level data. There were two distinct enterotypes: enterotype 1 (E1) with a high relative abundance of *Bacteroides*, and enterotype 2 (E2) with a high relative abundance of *Prevotella* (Fig. 2a–b). Although no significant differences were observed in UA, HDLC and their ratio between the E1 and E2 enterotypes (see Additional File 4), MaAsLin2 analysis showed that *Bacteroides* was in positive correlation with UA, *Prevotella* was in negative correlation with UA, and *Prevotella* was in positive correlation with HDLC (Fig. 2c).

Interestingly, the proportion of individuals with a blood stasis constitution was significantly higher in E1 than in E2, in both all samples and female samples only (Fig. 2d). In fact, only one female with the blood stasis constitution was found in the E2 enterotype group, and this individual was excluded when comparing female participants stratified by constitution and enterotype (BloodStasis_E1, Balanced_E1, Balanced_E2). As expected, BloodStasis_E1 had significantly higher UA and UHR but lower HDLC than the other two groups (Fig. 2e). Notably, Balanced_E1 showed intermediate values for these three parameters, with significant differences between these two Balanced subgroups. Finally, LEfSe was employed for the identification of the microbiota markers in the three subgroups (Fig. 2f). While the E1 enterotype was dominated by *Bacteroides*, markers of this taxon were enriched mainly in BloodStasis_E1, including *B. vulgatus*, *B. uniformis* and *B. thetaiotaomicron*, whereas Balanced_E1 had only one *Bacteroides* taxon marker *B. ovatus*. Balanced_E2, on the other hand, was characterized by markers of Prevotellaceae, such as *P. copri* and *Paraprevotella xylaniphila*. These results indicated that the distribution of signature gut microbes differed by constitution even within the same enterotype. Overall, two enterotypes associated with UA, HDLC and UHR were identified. The disproportionate prevalence of blood stasis constitution in *Bacteroides*-dominant E1 suggested some synergy between the microbial profiles and the three biochemical markers.

### Serum metabolomic analysis revealed subtle differences between females with blood stasis and those with a balanced constitution

**Fig. 3:**
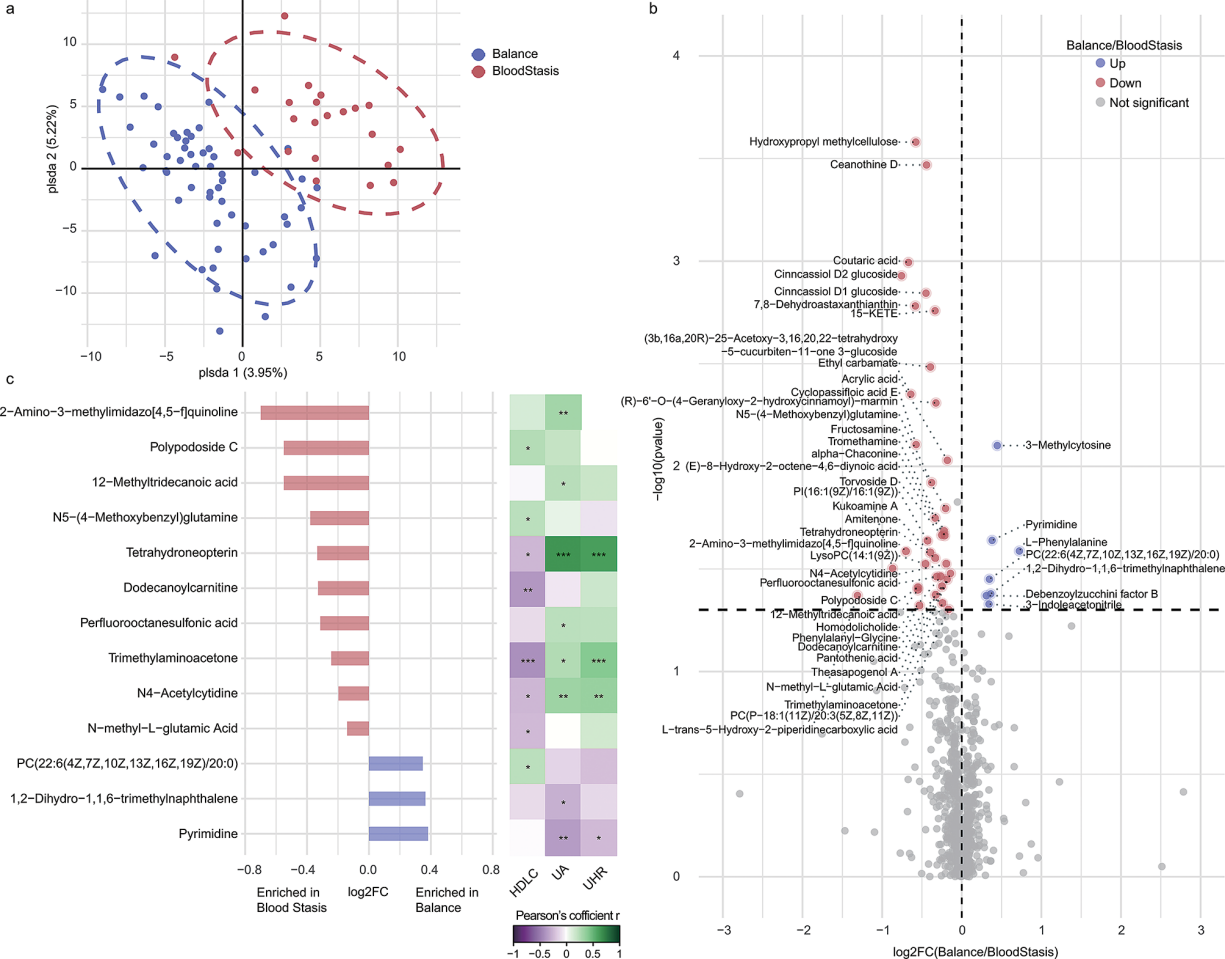
Results of serum metabolomic analysis in females. (**a**) Comparison of blood stasis (raspberry) and balance (medium slate blue) by PLS-DA. (**b**) Volcano plots show significantly differential metabolite involved in balance versus blood stasis. Each point represents a metabolite. Significant features of the metabolite markers based on the VIP > 1 and t-test *P* value < 0.05 were in raspberry (enriched in blood stasis) or medium slate blue (balance). (**c**) Differential metabolites related to UA, HDLC or UHR. Pearson correlation significance is indicated by: * *P* < 0.05, ** *P* < 0.01, *** *P* < 0.001

Metabolomic profiling revealed 19 differential serum metabolites between the blood stasis and the balanced groups (Additional File 1, Table S2). Among these 19 metabolites, none exhibited a fold change exceeding 2. and only three showed a fold change greater than 1.5 (Additional File 1, Table S2), indicating that the overall metabolic profiles were still relatively small. L-phenylalanine and hydrogen phosphate exhibited significantly higher in the balanced group, while PS (20:5(5Z,8Z,11Z,14Z,17Z)/18:1(9Z)) was higher in the blood stasis group (Additional File 1, Table S2). To eliminate the influence of sex, only female subjects were analyzed again. PLS-DA indicated a clear clustering between the blood stasis and balanced groups (Fig. 3a). A total of 44 differential metabolites were identified based on a VIP > 1 and a t-test *P* value < 0.05. The metabolites exhibiting the greatest changes in expression were L-trans-5-hydroxy-2-piperidinecarboxylic acid (higher in the blood stasis group) and L-phenylalanine (higher in the balanced group) (Fig. 3b). Correlation analysis showed that 13 of these metabolites were significantly associated with HDLC, UA or UHR (Fig. 3c). Serum metabolites with higher levels in the blood stasis group were predominantly in negative correlation with HDLC or positive correlation with UA. Notably, trimethylaminoacetone, tetrahydroneopterin, and N4-acetylcytidine were negatively correlated with HDLC and positively correlated with both UA and UHR. Among the metabolites higher in the balanced group, pyrimidine was significantly in negative correlation with UA and UHR, while PC(22:6(4Z,7Z,10Z,13Z,16Z,19Z)/20:0) was in positive correlation with HDLC, and 1,2-dihydro-1,1,6-trimethylnaphthalene was negatively correlated with UA. These results suggested that specific serum metabolites display distinct associations with HDLC, UA, and UHR in the blood stasis and balanced groups.

## Discussion

This study investigated the relationships between blood stasis constitution, a concept in TCM, and serum biochemical markers of metabolic health, such as UA, HDLC, and UHR. A notable predominance of blood stasis constitution was identified among females, with significantly fewer occurrences observed in males. This observation aligns with a recent research finding, suggesting that females were particularly predisposed to a blood stasis constitution due to physiological and hormonal factors such as menstruation, childbirth, and breast-feeding, which inherently affect blood flow and stasis [28]. That study further suggested lifestyle factors, emotional state, and certain environmental factors prevalent among women may contribute to this predisposition. These collective insights highlight the importance of considering sex-specific physiological and lifestyle factors in diagnosing and treating a blood stasis constitution. Further research is necessary to explore the complex interplay of these factors and to develop gender-specific preventative and therapeutic strategies.

The relationships among the gut microbiome, constitution, and UHR in female participants were also explored. It was first found that females with blood stasis constitution had significantly higher UA, lower HDLC, and higher UHR than females with balanced constitutions, suggesting a higher risk of metabolic abnormalities. UA level, HDLC level and UHR have been proposed as risk prediction indicators for metabolic syndrome [10, 11]. Targeted interventions for females with a blood stasis provide valuable insight for clinical practice.

The gut microbiome composition of the participants was clustered into two enterotypes (E1 and E2) based on the relative abundance of *Bacteroides* and *Prevotella*. This finding matches previous reports of similar enterotypes in different populations [29]. *Bacteroides* and *Prevotella* are two major genera of the human gut microbiome that have distinct metabolic capacities and ecological niches. *Bacteroides* species are more efficient at degrading complex polysaccharides and proteins, while *Prevotella* species are more efficient at fermenting simple sugars and producing short-chain fatty acids [30–32]. *Bacteroides* and *Prevotella* have also been shown to modulate host metabolism, immunity, and inflammation in different ways [33–36]. The associations between UA levels, HDLC levels, UHR, and the prevalence of a blood stasis constitution imply that the microbiome could potentially contribute to metabolic dysregulation in females. The distribution of signature gut microbes differs by constitution even within the same enterotype, indicating that constitution may be a finer-grained classification of the gut microbiome than enterotype. For example, within the E1 enterotype, the blood stasis group had more *Bacteroides* taxa markers than the balanced group, while within the E2 enterotype, the balanced group had more Prevotellaceae taxa markers than the blood stasis group. These results indicate that constitution may reflect the subtle differences in gut microbiome composition and function that are not captured by enterotype. Therefore, constitution may be helpful in stratifying individuals based on their gut microbiome profiles and metabolic health status.

Although there were dozens of differential metabolites between the female blood stasis and balanced groups, the fold changes in these metabolites between the groups were not substantial. Given that individuals in both groups were deemed healthy based on laboratory indicators according to definitions, this could indicate that the biological processes related to these metabolites are not markedly different between the two groups, or that the differences may be subtle and driven by other factors, such as individual variability or environmental influences. Additional investigation must be conducted to understand the underlying causes of these minor differences and their potential implications in the context of blood stasis and health conditions.

One of the differential metabolites between the blood stasis and balanced groups, L-phenylalanine, is an essential amino acid involved in various metabolic pathways [37–39]. Interestingly, a previous study by [40] found weak correlations between L-phenylalanine and total cholesterol, triglycerides, and LDLC. Additionally, there was a weak inverse correlation between L-phenylalanine and HDLC. In our study, a significant increase in L-phenylalanine levels was observed in the balanced group compared to the blood stasis group, consistent with the overall trend of higher HDLC and lower UA levels in the balanced group. Therefore, L-phenylalanine may play a role in modulating HDLC and UA levels with the blood stasis constitution. However, the relationship between L-phenylalanine and UA levels was not directly addressed in the previous study, and the causal direction and mechanism of the association between L-phenylalanine and lipid levels are still unclear. Additional assessments are warranted to elucidate the specific role of L-phenylalanine in metabolic health and its interaction with blood stasis constitution.

Apart from L-phenylalanine, several metabolites exhibited significant correlations with HDLC, UA, or UHR were also deserve attention. Previous studies have implicated some of these metabolites in metabolic pathways. For instance, trimethylaminoacetone is a derivative of carnitine [41], which mediates lipid metabolism by facilitating the transportation of long-chain fatty acids into the mitochondria for beta-oxidation [42, 43]. Carnitine is also a precursor of trimethylamine N-oxide, a pro-atherogenic metabolite linked to an elevated risk of cardiovascular events, including myocardial infarction, stroke, and death [44]. PC(22:6(4Z,7Z,10Z,13Z,16Z,19Z)/20:0) is a specific member of the phosphatidylcholine (PC) family, and alterations in PC levels have been linked to lipid metabolism disorders [45]. However, the precise role of PC(22:6(4Z,7Z,10Z,13Z,16Z,19Z)/20:0) in lipid metabolism has not been explicitly discussed. In addition, it was discovered that N4-acetylcytidine, pyrimidine, tetrahydroneopterin, and 1,2-dihydro-1,1,6-trimethylnaphthalene displayed strong correlations with lipid and UA metabolic markers, despite the lack of previous studies elucidating their relationship with these metabolic processes. This pattern implies that these metabolites may contribute to or protect against the metabolic dysregulation associated with a blood stasis constitution. Although further investigation into the specific mechanisms of action is warranted. These findings provide initial evidence that these metabolites could serve as novel markers or targets for modulating metabolic health risks in individuals with a blood stasis constitution.

In future work, larger sample sizes and longitudinal cohort studies will be planned to further research to explore the relationship between the blood stasis constitution and metabolic abnormalities. Research is also plannead in populations with diseases and/or with intervention to obtain a more comprehensive understanding of the characteristics of blood stasis constitution in screening, early warning, and intervention. These efforts will enhance the understanding of the mechanisms underlying the association between the blood stasis constitution and metabolic abnormalities, and the development of potential interventions to reduce the risk of metabolic abnormalities in individuals with blood stasis constitution.

## Study strengths and limitations

### Strengths

To our knowledge, this study comprehensively investigated, for the first time, the associations among a blood stasis constitution, gut microbiota, and serum metabolites by integrating metabolomic analysis, gut microbiota profiling, and constitutional assessments. Moreover, sex-specific analyses add granularity to the investigation, recognizing and addressing potential sex-specific variations.

### Limitations

First, a small sample size was used, and only healthy individuals from a single region in China were included. These findings may not extend to other populations or individuals with diseases. Second, the temporal relationship or causality between the blood stasis constitution and serum metabolic indicators was not assessed. Third, other potential influencing factors that may affect the serum levels of UA and HDLC, including variables such as diet, lifestyle, medication use, and genetic factors, were not measured.

## Conclusion

This investigation explored the intricate relationships among the blood stasis constitution, gut microbiota, and serum metabolites, specifically focusing on females. The identified associations between the blood stasis constitution and higher UA levels, lower HDLC levels, and elevated UHR indicate a potential metabolic risk. Moreover, identifying two distinct enterotypes based on gut microbiome composition underscores the complexity of these interactions. These findings are clinically relevant by shedding light on the early biological processes of metabolic deterioration from a TCM perspective.

### Electronic supplementary material

Below is the link to the electronic supplementary material.

Additional file 1: Table S1. Statistical table of clinical information for the two constitutions. Table S2. Differentially metabolites identified by PLSDA and t-test (All samples).

Additional file 2: Figure. Histogram showing the relative abundances of (a) Phylum, (b) Class, (c) Genus, and (d) Species in all samples except two individuals for whom fecal samples were not available.

Additional file 3: Alpha diversity (richness, Shannon index, Simpson index) between blood stasis and balance in (a) the whole cohort and (b) the females, respectively. No significant differences were observed for these indicators, using a significance level of t-test *P* value < 0.05.

Additional file 4: The boxplots of (b) UHR, (c) UA, and (d) HDLC between the two enterotypes. No significant differences were observed for these indicators, using a significance level of t-test *P* value < 0.05.

## Data Availability

The microbiome and metabolome datasets analyzed during the current study will be available in the China National GeneBank Sequence Archive (CNSA). (Because data has not been uploaded, there is no ID number. Data will be uploaded before publication.)

## Abbreviations

TCM: Traditional Chinese Medicine
UA: Uric acid
HDLC: High-density lipoprotein cholesterol
UHR: Uric acid to high-density lipoprotein cholesterol ratio
PCA: Principal component analysis
PLS-DA: Partial least squares method-discriminant analysis
VIP: Variable Importance in Projection
PC: Phosphatidylcholine
