# Supplementary material for: Uric acid, high density lipoprotein cholesterol levels and their ratio are related to microbial enterotypes and serum metabolites in females with a blood stasis constitution
Source: Lipids Health Dis. 2024 Mar 27;23:90. doi: 10.1186/s12944-024-02066-4 (PMC10976831; doi:10.1186/s12944-024-02066-4)
Supplement: Supplementary file 1 — Additional file 1: Table S1. Statistical table of clinical information for the two constitutions. Table S2. Differentially metabolites identified by PLSDA and t-test (All samples). [file 12944_2024_2066_MOESM1_ESM.docx]

Supplementary Table1. Statistical table of clinical information.

| **Characteristics** | **Balance**  (N=80) | **Blood stasis**  (N=24) | | ***P* value**^1^ |
| --- | --- | --- | --- | --- |
| **General information** |  |  | |  |
| Sex (F/M) | 55/25 | 22/2 | | **0.032**^1^ |
| Age (yrs) | 35±6 (80) | 34±6 (24) | | 0.5 |
| **Anthropometric markers** |  |  | |  |
| Height (cm) | 163.9±6.3 (73) | 161.7±4.8 (23) | | 0.085 |
| Weight (kg) | 61±11 (75) | 59±10 (22) | | 0.5 |
| Systolic blood pressure (SBP, mm Hg) | 117±10 (66) | 113±14 (15) | | 0.4 |
| Diastolic blood pressure (DBP, mm Hg) | 72±8 (66) | 70±6 (15) | | 0.4 |
|  |  |  | |  |
| **Fasting blood-glucose** |  |  | |  |
| Fasting blood glucose (GLU, mmol/L) | 5.28±0.86 (78) | 5.25±0.42 (23) | | 0.8 |
|  |  |  | |  |
| **Lipometabolism indicators** |  |  | |  |
| Total cholesterol (TC, mmol/L) | 4.88±0.76 (78) | 4.75±1.11 (23) | | 0.6 |
| Triglyceride (TG, mmol/L) | 1.15±0.66 (78) | 0.97±0.35 (23) | | 0.086 |
| high density lipoprotein  (HDLC, mmol/L) | 1.41±0.30 (78) | 1.38±0.27 (23) | | 0.7 |
| low-density lipoprotein  (LDLC, mmol/L) | 3.04±0.70 (78) | 2.97±1.03 (23) | | 0.7 |
|  |  |  | |  |
| **Inflammatory indicators** |  |  | |  |
| C-reactive protein (CRP, mg/L) | 1.17±1.94 (78) | 1.10±1.79 (23) | | 0.9 |
|  |  |  | |  |
| **Renal function index** |  |  | |  |
| Uric acid (UA, μmol/L) | 336±104 (78) | 336±87 (23) | | >0.9 |
| Urea (mmol/L) | 4.59±1.10 (78) | 4.48±0.86 (23) | | 0.6 |
| Creatinine (μmol/L) | 69±16 (78) | 63±11 (23) | | 0.050 |
| eGFR (ml/min/1.73 m^2^) | 107±14 (78) | 110±15 (23) | | 0.4 |
|  |  |  | |  |
| **Liver function test** |  |  | |  |
| Alanine transaminase (ALT, U/L) | 17±13 (78) | 13±7 (23) | | **0.029** |
| Aspartate transaminase (AST, U/L) | 18±6 (78) | 16±4 (23) | | 0.075 |
| AST/ALT | 1.26±0.46 (76) | 1.35±0.36 (21) | | 0.4 |
| Total protein (TP, g/L) | 75.2±4.1 (78) | 74.3±4.0 (23) | | 0.3 |
| Albumin (ALB, g/L) | 48.6±3.0 (78) | 48.3±2.9 ((23) | | 0.7 |
| Albumin/Globulin (ALB/GLB) | 1.86±0.31 (78) | 1.90±0.28 (23) | | 0.6 |
| Gamma-glutamyl transferase (GGT, U/L) | 22±20 (78) | 17±16 (23) | | 0.3 |
| Total bile acid (TBA, μmol/L) | 6.0±6.6 (78) | 7.2±13.9 (23) | | 0.7 |
|  |  |  | |  |
| **Whole blood cell analysis** |  |  | |  |
| White blood cell (WBC, 10^9^/L) | 6.21±1.58 (78) | 6.10±1.23 (23) | | 0.7 |
| Neutrophil (NEUT, 10^9^/L) | 3.51±1.23 (78) | 3.42±1.00 (23) | | 0.7 |
| Lymphocyte (LYM, 10^9^/L) | 2.11±0.59 (78) | 2.11±0.57 (23) | | >0.9 |
| Monocyte (MONO, 10^9^/L) | 0.41±0.13 (78) | 0.37±0.09 (23) | | 0.095 |
| Basophil (BASO, 10^9^/L) | 0.03±0.02 (78) | 0.04±0.02 (23) | | 0.311 |
| Eosinophil (EOSIN, 10^9^/L) | 0.15±0.11 (78) | 0.15±0.10 (23) | | 0.8 |
| Red blood cell (RBC, 10^12^/L) | 4.81±0.62 (78) | 4.63±0.47 (23) | | 0.1 |
| Hemoglobin (HB, g/L) | 139±17 (78) | 133±14 (23) | | 0.14 |
| Red blood cell distribution width  (RDW, %) | 12.68±1.47 (78) | 12.47±0.93 (23) | | 0.4 |
| Hematocrit (HCT, %) | 42.0±4.5 (78) | 40.3±3.8 (23) | | 0.082 |
| mean corpuscular hemoglobin  (MCH, pg) | 29.0±3.2 (78) | 29.0±3.5 (23) | | >0.9 |
| mean corpuscular volume (MCV, fl) | 88±7 (78) | 88±8 (23) | | >0.9 |
| mean platelet volume (MPV, fl) | 9.98±0.85 (77) | 10.51±1.06 (23) | | **0.035** |
| platelet hematocrit (PCT, %) | 0.27±0.05 (77) | 0.26±0.05 (23) | | 0.8 |
| platelet count (PLT, 10^9^/L) | 271±54 (78) | 254±48 (23) | | 0.2 |
| **thyroid function** |  |  | |  |
| free triiodothyronine (FT3, pmol/L) | 5.19±0.57 (78) | 4.98±0.46 (23) | | 0.077 |
| free thyroxine (FT4, pmol/L) | 15.46±2.01 (78) | 15.07±1.82 (23) | | 0.4 |
| **Cancer index** |  |  | |  |
| alpha fetoprotein (AFP, ng/mL) | 7.18±31.32 (78) | 2.10±1.42 (23) | | 0.2 |
| carcinoembryonic antigen  (CEA, ng/mL) | 1.72±1.03 (78) | 1.69±1.04 (23) | | 0.9 |
|  | | |  |  |

The information was summarized by mean±SD and number. Integer in parentheses represents a number of samples.

^1^Fisher's exact test was used to compare the differences among the two constitutions. *P*＜0.05 indicated statistically significant.

Supplementary Table 2. Differentially metabolites identified by plsda and t-test (All samples)

| **Metabolite** | **Balance**  **(mean)** | **Blood stasis**  **(mean)** | **ratio** | **logFC** | ***P* value** | **VIP** |
| --- | --- | --- | --- | --- | --- | --- |
| Hydroxypropyl methylcellulose | 0.0041 | 0.0052 | 0.7905 | -0.3391 | 0.0149 | 1.7717 |
| 2-Propylpyridine | 0.0519 | 0.045 | 1.1549 | 0.2078 | 0.046 | 1.5355 |
| 3-Methylcytosine | 0.0181 | 0.0134 | 1.3455 | 0.4282 | 0.007 | 2.5868 |
| Ethyl carbamate | 0.0633 | 0.082 | 0.7721 | -0.3732 | 0.0013 | 1.751 |
| Debenzoylzucchini factor B | 0.003 | 0.0027 | 1.1284 | 0.1743 | 0.027 | 1.1253 |
| Coutaric acid | 0.025 | 0.034 | 0.7346 | -0.4451 | 0.0168 | 1.8147 |
| PC(P-18:1(11Z)/20:3(5Z,8Z,11Z)) | 0.8034 | 0.918 | 0.8752 | -0.1924 | 0.0303 | 1.7817 |
| Tromethamine | 0.0402 | 0.0458 | 0.8775 | -0.1885 | 0.0418 | 1.2891 |
| PS(20:5(5Z,8Z,11Z,14Z,17Z)/18:1(9Z)) | 0.0865 | 0.1412 | 0.6124 | -0.7074 | 0.049 | 1.9054 |
| Ceanothine D | 0.0087 | 0.0103 | 0.8483 | -0.2373 | 0.0163 | 1.7291 |
| Cinncassiol D1 glucoside | 0.0139 | 0.0164 | 0.8458 | -0.2416 | 0.0261 | 1.7208 |
| PI(16:1(9Z)/16:1(9Z)) | 0.0487 | 0.0597 | 0.815 | -0.2951 | 0.0466 | 1.5852 |
| (3b,16a,20R)-25-Acetoxy-3,16,20,22-tetrahydroxy-5-cucurbiten-11-one-3-glucoside | 0.0083 | 0.0107 | 0.777 | -0.3641 | 0.0448 | 1.7298 |
| Cinncassiol D2 glucoside | 0.0164 | 0.0217 | 0.7548 | -0.4058 | 0.0486 | 1.664 |
| (E)-8-Hydroxy-2-octene-4,6-diynoic acid | 0.0027 | 0.0031 | 0.8547 | -0.2266 | 0.0098 | 1.7385 |
| Acrylic acid | 0.2662 | 0.2911 | 0.9142 | -0.1294 | 0.0212 | 1.5397 |
| L-Phenylalanine | 0.0974 | 0.0493 | 1.9767 | 0.9831 | 0.0019 | 1.9592 |
| Hydrogen phosphate | 0.1135 | 0.075 | 1.5122 | 0.5966 | 0.0156 | 1.4222 |
| 2-Amino-3-methylimidazo[4,5-f]quinoline | 0.0199 | 0.0276 | 0.722 | -0.47 | 0.0474 | 1.2362 |
